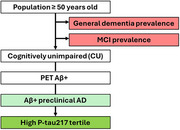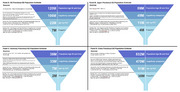# Estimating Preclinical Alzheimer's Disease Populations by *p*‐tau217 in the United States, Japan, Germany, and China

**DOI:** 10.1002/alz70860_102861

**Published:** 2025-12-23

**Authors:** Zachary Sheff, Nalin Payakachat, E. Jolanda Muenzel, Roy Yaari, Karen Chilcott Holdridge, Dulanji Kuruppu, Julie Chandler

**Affiliations:** ^1^ Eli Lilly & Company, Indianapolis, IN, USA

## Abstract

**Background:**

Preclinical Alzheimer's disease (AD) is the earliest clinical stage of AD in which individuals have biomarker evidence of AD but are cognitively unimpaired. Current amyloid detection via PET scans or CSF collection is not typically performed in cognitively unimpaired individuals outside of research settings. Blood‐based biomarkers (BBMs) are an emerging minimally invasive alternative that potentially offers scalability and low cost. This study estimates numbers of individuals with preclinical AD aged 50+ who may be at the greatest risk for future cognitive decline in United States, Japan, Germany, and China, based on recent published population estimates and plasma *p*‐tau217 data.

**Methods:**

Starting from country‐specific census data, prevalence estimates for general dementia, mild cognitive impairment, and amyloid PET positivity from the literature were applied to estimate preclinical AD population size (Figure 1). Recent literature demonstrated that among cognitively unimpaired individuals with elevated amyloid, plasma *p*‐tau217 levels in the top tertile had the highest rate of clinical progression, compared to those in lower tertiles. We estimated the size of the preclinical AD population at greatest risk for cognitive decline by taking one‐third of the preclinical AD population estimated by amyloid PET.

**Results:**

Estimated preclinical AD population sizes based on amyloid PET among adults aged 50+ were 22.0 million (18.3%) in the United States, 11.2 million (19.0%) in Japan, 7.0 million (18.5%) in Germany, and 90.7 million (17.0%) in China (Table 1). Assuming one‐third of the preclinical AD population is at greatest risk for cognitive decline based on top tertile plasma *p*‐tau217 levels, the estimated population sizes were reduced to 7.3 million (6.1%) in the US, 3.7 million (6.3%) in Japan, 2.3 million (6.2%) in Germany, and 30.2 million (5.7%) in China (Figure 2).

**Conclusions:**

Given the estimated scale of the preclinical AD population, BBMs like plasma *p*‐tau217 may help reduce the burden on healthcare system infrastructure and budgets by identifying those at greatest risk of progression, who may be benefit from future treatments. Data on prevalence of plasma *p*‐tau positivity in the preclinical AD population is needed for robust estimates that will help determine economic impact of future treatment.